# Enteric Bacteria and Parasites with Pathogenic Potential in Individuals of the Colombian Indigenous Tribe Kogui

**DOI:** 10.3390/microorganisms10091862

**Published:** 2022-09-17

**Authors:** Simone Kann, Gustavo Concha, Thomas Köller, Juliane Alker, Ulrich Schotte, Andreas Hahn, Hagen Frickmann, Philipp Warnke

**Affiliations:** 1 Missionsärztliches Institut, 97074 Würzburg, Germany; 2 Organization Wiwa Yugumaiun Bunkauanarrua Tayrona (OWYBT), Department Health Advocacy, Valledupar 2000001, Colombia; 3 Institute for Medical Microbiology, Virology and Hygiene, University Medicine Rostock, 18057 Rostock, Germany; 4 Department A-Veterinary Medicine, Central Institute of the Bundeswehr Medical Service Kiel, 24119 Kronshagen, Germany; 5 Department of Microbiology and Hospital Hygiene, Bundeswehr Hospital Hamburg, 20359 Hamburg, Germany

**Keywords:** Kogui, indigenous, Colombia, gastroenteritis, pathogens, hygiene, real-time PCR, screening

## Abstract

The Kogui tribe is an indigenous population living in Colombia. The prevalence values of some enteric bacteria, parasites and microsporidia in Kogui stool samples (*n =* 192) were assessed by real-time polymerase chain reaction (PCR). Thus, genus- or species-specifically recorded positivity rates among the Kogui community were assessed. Protozoa were the leading microorganisms in the stool samples of the Kogui, with an average of 1.5 pathogens per sample, followed by bacteria, with 0.6 pathogens per samples and helminths, with 0.3 pathogens per sample. Microsporidia were not detected. Thereby, the majority of detected protozoa comprised species with questionable etiological relevance such as *Blastocystis hominis* (*n =* 173) and *Dientamoeba fragilis* (*n =* 44), but also a considerable proportion of *Giardia duodenalis* (*n =* 71). *Cryptosporidium* spp., in contrast, was found in a single instance only. The majority of recorded bacteria were *Campylobacter* spp., with a strikingly high proportion of 50% (*n =* 96), followed by *Shigella* spp./enteroinvasive *E. coli* (EIEC) (*n =* 14) and *Aeromonas* spp. (*n* = 4). The quantitatively most important detected helminths were *Ascaris* spp. (*n* = 15), *Hymenolepis* spp. (*n* = 14) and *Trichuris trichiura* (*n* = 12), followed by *Necator americanus* (*n* = 6), *Taenia* spp. (*n* = 3) and *Strongyloides stercoralis* (*n* = 3) in descending order of abundance. As expected, the Kogui people’s living conditions comprising poverty, lack of access to clean water and simple housing favor a high number of gastrointestinal infections. Preventive approaches are needed to reduce their risk of infection.

## 1. Introduction

Kogui (also addressed as “Kogi” in some publications) communities are indigenous tribes living under resource-poor conditions in remote areas of the Sierra Nevada de Santa Marta (SNSM) in the north-east of Colombia. Medical and epidemiological knowledge on this indigenous population is scarce. Previous research on Kogui communities was focused on ethnological aspects [1], genetic association studies in comparison to other indigenous and non-indigenous populations [2,3,4] as well as the distribution of their molecular immunological determinants such as human leukocyte antigens (HLA) and major histocompatibility classes [5,6].

Other than for the Kogui people, however, extensive epidemiological studies on infectious disease threats have been conducted with indigenous Wiwa populations living in the same geographic region in the previous few years. Those analyses comprised systemic infections such as Chagas disease [7], leptospirosis [8], toxocariasis [9] and COVID-19 [10]. As indicated by an assessment on the general health conditions in the local Wiwa communities [11], infectious gastroenteritis and diarrhea have been identified as quantitatively dominating health concerns. Several studies on infectious gastroenteritis in Wiwa populations have indicated high pathogen loads with enteropathogenic bacteria and parasites [12,13,14,15].

In those previous works [12,13,14,15], lacking enforcement of hygiene precautions associated with living under resource-limited conditions and a lack of knowledge had been blamed for the high prevalence of gastroenteric pathogens in the Wiwa population. The Kogui people, of note, live under similar conditions and are located not so far apart from the Wiwa communities. Still, there are some differences between the tribes in lifestyle and traditions, e.g., the Kogui people live close to San Jose as the next biggest city, which is reachable within one hour walking distance. In contrast, the Wiwa villages are spread out in the SNSM and, depending on the locality of the village, 1–7 h is the walking distance to the next equipped health point. The Kogui community has a permanently equipped health point in San Jose available, where two nurses are in charge. Although both tribes live close together with their livestock, the Kogui people keep their animals within fences and feed them actively, while the Wiwa people let the animals take care of themselves, with the consequence that, e.g., chicken run in the houses and even the kitchens, resulting in low general hygiene. Living conditions, such as the housing, are similar and access to fresh water, which derives from unprotected wells or the river, is the same. However, due to the location of the Kogui villages nearer to the water sources, the water quality might be better than in the Wiwa villages.

To close the knowledge gap on the prevalence of gastrointestinal infections in the Colombian Kogui populations, a cross-sectional study was performed. The hypothesis was that Kogui populations are also highly affected, similar to what is shown for the Wiwa people in previous reports [12,13,14,15]. To get a rough impression of the regional background infection rate, a small group of non-indigenous Colombians from the same geographic region but within a well-developed urban setting was also assessed during the study period.

## 2. Materials and Methods

### 2.1. Study Design and Study Populations

**Figure 1:**
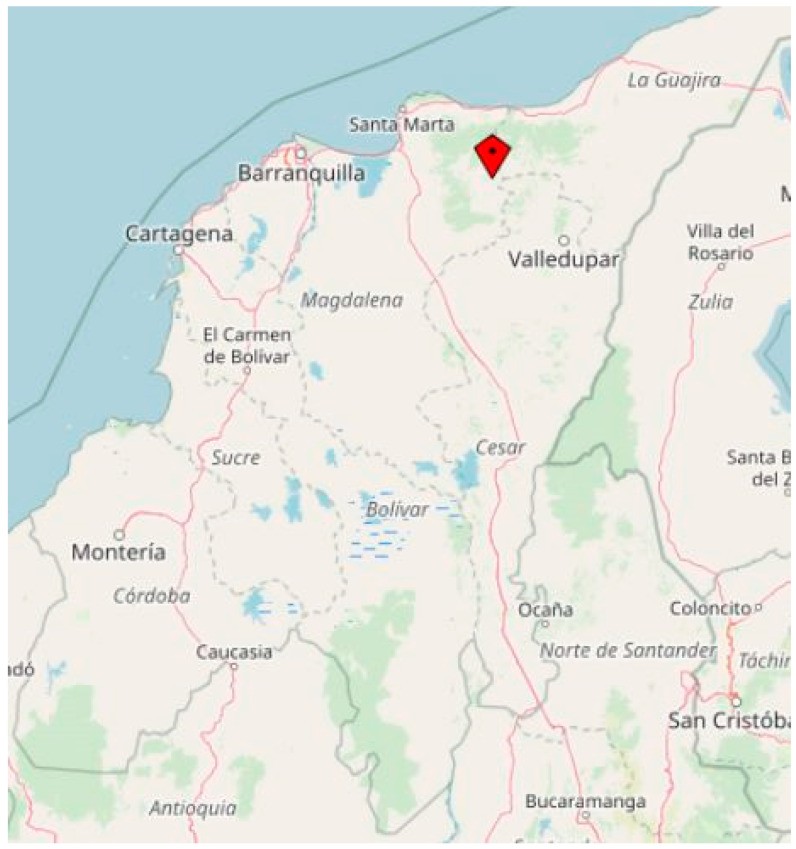
Northern coastline of Colombia. The study site Sierra Nevada de Santa Marta (SNSM) is marked with a red square. The figure was created with OpenStreetMap (https://www.openstreetmap.de/ last accessed on 3 September 2022).

The study was designed as a cross-sectional epidemiological assessment of stool samples collected from indigenous Kogui people between November 2020 and March 2022 in the Sierra Nevada de Santa Marta (SNSM) in the north-east of Colombia (Figure 1). To get an impression of the background infection pressure, a small number of stool samples from non-indigenous Colombians living in an urban setting of the nearby city Valledupar was collected to get a rough impression of the background infection pressure in October 2021. Next to the collection of stool samples, general information on age, sex and living place were recorded as well. Due to the known unreliability of anamnestic information of gastrointestinal symptoms in Colombian indigenous populations based on a reporting bias because of social undesirability as reported elsewhere [12,13,14,15], no clinical details on the study participants are provided and the study was focused on prevalence assessment only.

### 2.2. Laboratory Techniques

After sampling in Colombia, all stool samples had been stored at −20 °C. In detail, non-fixed stool samples in small sampling containers were put in a cooling box for transport within the first hour after sampling and subjected to freezing at −20 °C on the day of sampling. Shipment on dry ice to Germany was conducted by World Courier (Frankfurt, Germany), following the national and flight associated requirements. Afterwards, storage was performed at −80 °C until nucleic acid extraction. Subsequently, the same storage conditions applied to the eluates after nucleic acid extraction. Between sampling and PCR analysis, there were average time periods of about 3 months.

Nucleic acid extraction was performed using the automated Nimbus extractor (SeeGene, Seoul, Korea) in line with the manufacturer’s instructions. Subsequently, the commercial real-time PCR assays Allplex GI-Bacteria(I), Allplex GI-Parasite and Allplex GI-Helminth(I) (SeeGene, Seoul, Korea) were run on a CFX96 qPCR instrument (Bio-Rad Laboratories, Inc., Hercules, CA, USA) and analysed by applying the SeeGene Viewer software, again according to the manufacturer’s instructions within the CE-IVD label in a laboratory accredited according to DIN EN ISO 15189. Details on the diagnostic accuracy of those assays have been published elsewhere [16,17]. In short, the assays target fungi (microsporidia), helminths (*Strongyloides* spp., *Hymenolepis* spp., *Ascaris* spp., *Taenia* spp., *Trichuris trichiura*, *Ancylostoma* spp., *Enterobius vermicularis*, *Necator americanus*), protozoa (*Blastocystis hominis*, *Giardia duodenalis*, *Dientamoeba fragilis*, *Entamoeba histolytica*, *Cyclospora cayetanensis*, *Cryptosporidium* spp.) and bacteria (*Shigella* spp./enteroinvasive *Escherichia coli* (EIEC) (without further discrimination of both), *Campylobacter* spp., *Yersinia enterocolitica*, *Vibrio* spp., *Clostridioides difficile* toxin B, *Aeromonas* spp., *Salmonella* spp.). High quality figures of the assessed parasites for interested readers are provided by the United States Centers for Diseases Control and Prevention (https://www.cdc.gov/dpdx/az.html last accessed on 3 September 2022). Cycle threshold (Ct) values were recorded to allow semi-quantification of the pathogen loads.

### 2.3. Statistical Assessments

In line with the study design, the data were just descriptively presented without a statistical workup.

### 2.4. Ethical Clearance

Ethical clearance for the assessment was provided by the Institutional Ethic Committee for Investigation, Bogota, Colombia, (Acta no. 2019-4). An informed consent form was signed by the study participants or the legal guardian of a child. The results of the analysis were given and explained to the participants by a doctor, and therapeutic interventions were initiated, if applicable. The study was conducted in line with the Declaration of Helsinki and all its amendments.

## 3. Results

### 3.1. Study Population

A total of 192 stool samples from Kogui participants were collected for the assessment between November 2020 and March 2022. The mean age (±standard deviation (SD)) of the study participants was 26.5 (±18.5) years, the female:male ratio was 35.4:64.6. The assessed Kogui settlements (in brackets: number (n) of participants recruited for the study) comprised Avingüe (*n* = 22), Chenducua (*n* = 25), Mamangueka (*n* = 14), Marwamake (*n* = 66), Pueblo Hernández (*n* = 17), Surumuke (*n* = 7) and Sarachui (*n* = 41); all villages belong to the Cesar Department. The small group of 43 non-indigenous Colombians from the intermediate-sized town Valledupar (Cesar Department) sampled in October 2021 showed a mean age (±SD) of 37.8 (±19.6) years and a female:male ratio of 58.1:41.9.

### 3.2. Pathogen Detections in Stool Samples of the Assessed Kogui Population

As detailed in Table 1, protozoa were the leading microorganisms in the Kogui people’s stool samples, with an average of 1.5 pathogens per sample, followed by bacteria, with 0.6 pathogens per sample and helminths, with 0.3 pathogens per sample. Microsporidia were not detected. Going a little more into detail, the majority of the detected protozoa comprised species with questionable etiological relevance, such as *B. hominis* and *D. fragilis,* but also a considerable proportion of *G. duodenalis*. *Cryptosporidium* spp., in contrast, was found in a single instance only. The majority of recorded bacteria were *Campylobacter* spp., with a strikingly high proportion of 50%, followed by *Shigella* spp./enteroinvasive *E. coli* (EIEC) and *Aeromonas* spp. The quantitatively most important helminths were *Ascaris* spp., *Hymenolepis* spp. and *T. trichiura*, followed by *N. americanus*, *Taenia* spp. and *S. stercoralis* in descending order of occurrence. Focusing on the semi-quantification based on the cycle threshold (Ct) values, high Ct values close to 40 suggested low numbers of DNA copies for both helminths and bacteria in the stool samples, while considerably lower Ct values suggestive of a higher pathogen density were recorded for the detected protozoa (details in Table 1).

Focusing on the infectious background noise as detectable in the group of the 43 non-indigenous Colombians, pathogenic bacteria and helminths were detected at an average likeliness of <0.1 per sample, while the average likeliness of being positive for an enteric protozoon was about 0.5 per sample (details in Table 1). Recorded bacterial and helminth findings were associated with high cycle threshold (Ct) values, indicating very low quantities of abundant pathogen DNA, as they may occur in the case of residual persistence of traces of pathogen DNA from previous, already cured, infections. Another situation was observed for the protozoa. Although the single detected *G. duodenalis* infection was associated with a moderate Ct value, which was still higher than the average measured in the Kogui people, the recorded Ct values for the 18 *B. hominis*-positive samples and for the 3 *D. fragilis*-positive samples suggested considerable parasite density in the stool samples and were relevantly lower than observed for the positive stool samples collected from the Kogui people (see Table 1 for comparison). Again, microsporidia were not detected.

## 4. Discussion

In the Sierra Nevada de Santa Marta in the north-east of Colombia, indigenous tribes such as the Wiwa and the Kogui live under very resource-limited conditions. As their neighbor tribe Kogui shares similarly poor living conditions like the Wiwa people, not surprisingly, the finding of high enteric pathogen prevalence values as reported for the Wiwa communities previously [12,13,15] could be confirmed for the stool samples from the Kogui people. Interestingly, however, a rather high background noise of protozoan infections was also seen in local non-indigenous Colombians living in a local urban setting; this regional baseline infection rate is to be considered when interpreting the findings. Nevertheless, the positive results as detected in the Kogui samples still quantitatively dominated by far. In particular, a strikingly high prevalence of *Campylobacter* spp. in the Kogui samples was quite obvious. This might have arisen either from a local outbreak or was caused by an overall increased abundance in this indigenous population, because such high proportions of positivity for *Campylobacter* spp. have not been described for the Wiwa population so far [12,13,15].

The *high Campylobacter* spp.-associated infection rate is particularly surprising, because *Campylobacter* spp.-infections are considered to be livestock associated. Therefore, at least a slightly better situation in the Kogui villages should have resulted from the differences in the handling of livestock between the indigenous communities of the Wiwa people and the Kogui people, as the Kogui people keep their livestock within fences so that the animals live apart from their houses. Furthermore, continuous medical care in the form of a permanently equipped health point in the nearby village, with possibilities to buy medication, to reach a hospital soon by car or motorcycle, etc., might have been expected to lead to a better general health situation in the Kogui population. However, at least regarding the carriage of intestinal pathogens, the Kogui people were shown to be severely affected as well.

As an interesting side effect of this assessment, the few assessed non-indigenous Colombians from Valledupar expectedly showed a lower hygiene-related colonization prevalence with *B. hominis* and *D. fragilis*, but associated with higher parasite loads as expressed by the lower measured cycle threshold values. This, and the confirmation that Kogui populations have considerably higher infection risks than the non-indigenous Colombians from Valledupar, might be associated with adaption processes leading to some sort of semi-immunity and associated immunological pathogen control in the indigenous people. The recorded high Ct values suggesting low pathogen loads for the most assessed pathogens in the stool samples of the Kogui people point in a similar direction. Although the data provided in this study are insufficient to prove this hypothesis, they are in line with previous findings from military deployments to areas with poor enforcement of hygiene precautions, where reduced frequencies of gastrointestinal disease in later stages of deployments were observed in line with presumed adaptation processes [18].

Due to the inconsistent documentation of gastrointestinal symptoms, which are rarely complained of by local indigenous people, because such complaints are socially discouraged in their communities and symptoms are generally very common, no association between pathogen findings and clinical symptoms was attempted. As known from previous assessments [19,20], colonization and infection are difficult to distinguish in high prevalence settings for gastroenteric pathogens and, thus, no respective discrimination is attempted here.

The major limitation of this study is that, due to limited funding options, only a certain number of participants could be included in the cross-sectional assessment, allowing only a rough estimation of prevalence. This is particularly the case for rarely occurring pathogens. Secondly, and for the same reason, the assessment had to be performed without a real negative control group, as only a few non-indigenous Colombians could be assessed to allow at least a rough estimation of the local infection pressure in an urban setting. Thirdly, the applied PCR-based diagnostic strategy has a number of admitted limitations. Thus, the applied Allplex helminth panel lacks the parameter *Schistosoma* spp., and the superior diagnostic accuracy of PCR compared with traditional microscopy is, admittedly, much less well established for helminths compared with protozoa as summarized elsewhere [21], and its standardization still leaves room for improvement in spite of recent proceedings such as the availability of international laboratory assessment schemes for helminth PCR [22]. In 2021, a French assessment [23] indicated higher detection thresholds of the Allplex helminth PCR assay compared with microscopy, an issue that particularly affected helminth species with hard eggshells and robust cuticula cells. In this French study [23], the problem of false negative PCR results could be improved by applying a more robust bead beating-based nucleic acid extraction scheme compared with the procedure within the CE-IVD-label provided by the manufacturer. In our hands, as reported elsewhere [16,24], the Allplex helminth assays showed comparable results to molecular competitor approaches when applied from the same DNA templates [16], combined with high diagnostic specificity [24] next to low inter- and intra-assay variance [24]. Further, bead beating-based nucleic acid extraction provided only moderate shifts of the measured cycle threshold values in a species-depending way in our previous assessments with the Allplex assays [24], as well as with other real-time PCRs targeting helminths, protozoa and bacteria [25]. Based on the available evidence and on our experience with the assays, we decided to accept a potentially higher detection threshold of some of the applied Allplex real-time PCRs, (a) compared with microscopy for helminth eggs and larvae [23] and (b) compared with applying more robust nucleic acid extraction schemes [23,24,25] outside the assays’ CE-IVD label for the benefits of (c) high specificity and reproducibility of the assay results [24], as well as (d) a high degree of standardization in the case of in-label application of the CE-IVD labelled assays in the accredited diagnostic laboratory. This decision, admittedly, implies that some individuals with parasite DNA density in stool below the Allplex assays’ detection threshold will likely have gone undetected; thus, the results presented here might even underestimate the true prevalence.

## 5. Conclusions

In spite of the above-mentioned limitations, this cross-sectional study on enteric pathogens in the Kogui communities has shown that these indigenous people are highly affected by infections with enteric microorganisms. As a side finding, immunological adaptation processes may intervene with pathogen loads as suggested by the lower quantities of *B. hominis* and *D. fragilis* in the gut of the indigenous people compared with the non-indigenous Colombians living in urban settings.

## Figures and Tables

**Table 1:** Number and proportions of positive test results in the assessed Kogui communities (*n* = 192) and non-indigenous Colombians from Valledupar (*n* = 43) as well as semi-quantification as indicated by cycle threshold (Ct) values.

	Kogui			Non-Indigenous Colombians		
	Total Number of Positive Results n/n	Proportion of Positive Results	Recorded Cycle Threshold (Ct) Values in Real-Time PCR (±Standard Deviation SD).	Total Number of Positive Results n/n	Proportion of Positive Results	Recorded Cycle Threshold (Ct) Values in Real-Time PCR (±Standard Deviation SD).
Fungi						
Microsporidia	0/192	0%	n.a.	0/43	0%	n.a.
Helminths						
*Strongyloides* spp.	3/192	1.6%	40.5 (±4.7)	2/43	4.7%	42.2 (±0.9)
*Hymenolepis* spp.	14/192	7.3%	38.7 (±2.9)	0/43	0%	n.a.
*Ascaris* spp.	15/192	7.8%	39.8 (±1.7)	0/43	0%	n.a.
*Taenia* spp.	4/192	2.1%	39.1 (±3.2)	0/43	0%	n.a.
*T. trichiura*	12/192	6.3%	39.1 (±1.7)	0/43	0%	n.a.
*Ancylostoma* spp.	0/192	0%	n.a.	0/43	0%	n.a.
*E. vermicularis*	0/192	0%	n.a.	0/43	0%	n.a.
*N. americanus*	6/192	3.1%	38.3 (±3.2)	0/43	0%	n.a.
Protozoa						
*B. hominis*	173/192	90.1%	31.2 (±4.4)	18/43	41.9%	29.1 (±3.6)
*G. duodenalis*	71/192	37.0%	31.4 (±4.4)	1/43	2.3%	33.5
*D. fragilis*	44/192	22.9%	36.3 (±4.2)	3/43	7.0%	29.2 (±4.3)
*E. histolytica*	0/192	0%	n.a.	0/43	0%	n.a.
*C. cayetanensis*	0/192	0%	n.a.	0/43	0%	n.a.
*Cryptosporidium* spp.	1/192	0.5%	40.3 (-)	0/43	0%	n.a.
Bacteria						
*Shigella* spp./EIEC	14/192	7.3%	42.1 (±3.7)			
*Campylobacter* spp.	96/192	50.0%	37.4 (±3.1)	2/43	4.7%	40.1 (±1.0)
*Y. enterocolitica*	0/192	0%	n.a.	0/43	0%	n.a.
*Vibrio* spp.	0/192	0%	n.a.	0/43	0%	n.a.
*C. difficile* toxin B	0/192	0%	n.a.	0/43	0%	n.a.
*Aeromonas* spp.	4/192	2.1%	43.2 (±1.7)	0/43	0%	n.a.
*Salmonella* spp.	0/192	0%	n.a.	1/43	2.3%	43.4 (-)

n.a.—not assessed, EIEC—enteroinvasive *Escherichia coli*.

## Data Availability

All relevant data are provided in the manuscript. Raw data can be made available at reasonable request.
